# Interplay between coronavirus, a cytoplasmic RNA virus, and nonsense-mediated mRNA decay pathway

**DOI:** 10.1073/pnas.1811675115

**Published:** 2018-10-08

**Authors:** Masami Wada, Kumari G. Lokugamage, Keisuke Nakagawa, Krishna Narayanan, Shinji Makino

**Affiliations:** ^a^Department of Microbiology and Immunology, The University of Texas Medical Branch, Galveston, TX 77555-1019;; ^b^Center for Biodefense and Emerging Infectious Diseases, The University of Texas Medical Branch, Galveston, TX 77555-1019;; ^c^Center for Tropical Diseases, The University of Texas Medical Branch, Galveston, TX 77555-1019;; ^d^Sealy Center for Vaccine Development, The University of Texas Medical Branch, Galveston, TX 77555-1019;; ^e^Institute for Human Infections and Immunity, The University of Texas Medical Branch, Galveston, TX 77555-1019

**Keywords:** nonsense-mediated mRNA decay, cytoplasmic RNA virus, coronavirus, inhibition of NMD, long 3′ UTR

## Abstract

Coronaviruses (CoVs), including severe acute respiratory syndrome CoV and Middle East respiratory syndrome CoV, are enveloped RNA viruses that carry a large positive-sense single-stranded RNA genome and cause a variety of diseases in humans and domestic animals. Very little is known about the host pathways that regulate the stability of CoV mRNAs, which carry some unusual features. Nonsense-mediated decay (NMD) is a eukaryotic RNA surveillance pathway that detects mRNAs harboring aberrant features and targets them for degradation. Although CoV mRNAs are of cytoplasmic origin, the presence of several NMD-inducing features (including multiple ORFs with internal termination codons that create a long 3′ untranslated region) in CoV mRNAs led us to explore the interplay between the NMD pathway and CoVs. Our study using murine hepatitis virus as a model CoV showed that CoV mRNAs are recognized by the NMD pathway as a substrate, resulting in their degradation. Furthermore, CoV replication induced the inhibition of the NMD pathway, and N protein (a viral structural protein) had an NMD inhibitory function that protected viral mRNAs from rapid decay. Our data further suggest that the NMD pathway interferes with optimal viral replication by degrading viral mRNAs early in infection, before sufficient accumulation of N protein. Our study presents clear evidence for the biological importance of the NMD pathway in controlling the stability of mRNAs and the efficiency of replication of a cytoplasmic RNA virus.

## Significance

Coronaviruses (CoVs) are important pathogens for humans and domestic animals. The development of effective countermeasures against CoVs requires an understanding of the host pathways that regulate viral gene expression and the viral subversion mechanisms. However, little is known about how the stability of viral mRNAs is controlled. We show that the nonsense-mediated decay (NMD) pathway, which primarily targets aberrant cellular mRNAs for degradation, also induced the degradation of CoV mRNAs that are of cytoplasmic origin. Our study further suggests the importance of CoV-induced inhibition of the NMD pathway, mediated by a viral protein, for efficient CoV replication. The present study highlights an interplay between the NMD pathway and CoVs that modulates viral replication by controlling the stability of viral mRNAs.

Coronaviruses (CoVs) cause a variety of diseases in humans and domestic animals. Most human CoVs usually cause mild to moderate respiratory infections, with the exception of severe acute respiratory syndrome CoV (SARS-CoV) and Middle East respiratory syndrome CoV (MERS-CoV) that cause serious respiratory illness in humans and represent a major public health threat with the potential to inflict massive economic losses (1–5). Currently, there are no approved vaccines and therapeutic agents against human CoVs. Studies that lead to a comprehensive understanding of CoV gene expression strategies and host interactions will provide the necessary knowledge required for the development of new and effective measures to control CoV replication.

CoVs belong to the order Nidovirales, in the family *Coronaviridae*, and are currently classified into four genera: alpha, beta, gamma, and delta CoVs. CoV is an enveloped virus that carries a large (∼30-kb) positive-sense RNA genome, which is structurally polycistronic, containing multiple open reading frames (ORFs) (6) (*SI Appendix*, Fig. S1). CoV particles carry a helical nucleocapsid, which is a complex of the viral genomic RNA and the nucleocapsid protein, N, enclosed in an envelope composed of the viral envelope proteins, S, M, and E. After infection, the genomic RNA is released into the cytoplasm and is translated to produce two large polyproteins encoded in gene 1, which occupies the 5′ two-thirds of the genome with two partially overlapping ORFs (*SI Appendix*, Fig. S1). The two polyproteins are processed by viral proteases to generate 15 or 16 nonstructural proteins, most of which are required for viral RNA synthesis (7). In addition to mRNA 1 (the intracellular form of genomic RNA), several subgenomic mRNAs are synthesized in infected cells (*SI Appendix*, Fig. S1). These subgenomic mRNAs encode viral structural proteins and accessory proteins, the latter of which are not essential for virus replication in cell culture but play a role in viral pathogenicity (8, 9). CoV mRNAs have a common 3′ end, constituting a 3′-coterminal nested-set structure; the 5′ end of all CoV mRNAs carry a common ∼70-nt leader sequence (*SI Appendix*, Fig. S1) (6). Accordingly, most of the CoV mRNAs, except for the smallest subgenomic mRNA, have multiple ORFs. Because only the 5′-most ORF in viral mRNAs is, in principle, used for translation, most of the CoV mRNAs have a long 3′ untranslated region (UTR); in the case of genomic RNA, the length of the 3′ UTR is ∼10 kb (*SI Appendix*, Fig. S1). Although steady progress has been made in understanding CoV gene expression strategies, there are still considerable gaps in our knowledge about the CoV–host interactions involved in the regulation of viral mRNA stability and viral gene expression.

Nonsense-mediated decay (NMD) is a eukaryotic RNA surveillance pathway that detects mRNAs harboring aberrant features [e.g., premature termination codon (PTC)], and targets them for degradation (10). NMD can occur on mRNAs during the pioneer round of translation as well as during the eIF4F-initiated steady-state translation (11, 12). PTC recognition is dependent on a protein complex called the exon junction complex (EJC), which is deposited on mRNAs during splicing. During the translation of normal transcripts, the elongating ribosome removes the EJCs located within the ORFs. In aberrant transcripts carrying PTC upstream of the last exon, EJCs remain bound to the mRNA after translation termination at the PTC, which in turn is recognized by the NMD effectors and other proteins, activating the NMD pathway. UPF1 (the principal orchestrator of NMD) and SMG1 interact with the peptide-release factors, which are associated with the stalled ribosome, at the PTC. Subsequently, UPF1 interacts with UPF2 at the EJC, which triggers UPF1 phosphorylation by SMG1 and the dissociation of the stalled ribosome with the peptide-release factors. The phosphorylated UPF1 recruits SMG6 and a complex of SMG5 and SMG7. SMG6 induces an endonucleolytic RNA cleavage and SMG5–SMG7 induces mRNA decay (10). In addition to PTCs, other NMD-inducing features on mRNAs include upstream ORFs in the 5′ UTR, introns in the 3′ UTR, multiple ORFs with internal termination codons in a single mRNA, and long 3′ UTRs (10, 13, 14). Also, an EJC-independent NMD pathway has been identified, in which UPF1 binds to the 3′ UTRs of mRNAs in the absence of an exon–exon junction and is activated probably by interacting with cytoplasmic EJC components or EJC components stably associated with 3′ UTRs (15).

The role of NMD as a cell-intrinsic antiviral defense mechanism against cytoplasmic RNA viruses harboring genomes with recognizable NMD-activating features has gained traction as a novel research area (13, 16, 17). However, our understanding of the interplay between NMD and cytoplasmic RNA viruses is still in its infancy, as only a limited number of studies have addressed this area of research (13, 16, 17). CoV mRNAs have several NMD-inducing features, including multiple ORFs with internal termination codons that create a long 3′ UTR (*SI Appendix*, Fig. S1), which could predispose them to recognition by the NMD pathway. The presence of the NMD-activating features in CoV mRNAs led us to test the hypotheses that the NMD pathway recognizes and degrades CoV mRNAs and that CoV has developed a strategy to suppress NMD to protect viral mRNAs from degradation. Our present study supports these hypotheses and reveals the role of N protein as an NMD inhibitor. Furthermore, our data suggests that the virus-induced inhibition of the NMD pathway is important for the protection of viral mRNAs from rapid decay, thereby facilitating efficient virus replication. Our study has identified, in a cytoplasmic RNA virus, a viral protein with an NMD inhibitory function that plays a role in protecting viral mRNAs from rapid decay. Except for retroviruses, whose mRNAs are synthesized in the nucleus and often involves splicing, our study represents a direct demonstration of the recognition and targeting of mRNAs of a cytoplasmic RNA virus by the NMD pathway, highlighting the biological importance of the virus-induced inhibition of the NMD pathway for efficient virus replication.

## Results

### MHV Genomic RNA Is a Target of the NMD Pathway, and Its Inhibition Enhances MHV Replication from Transfected MHV Genomic RNA.

**Fig. 1.**
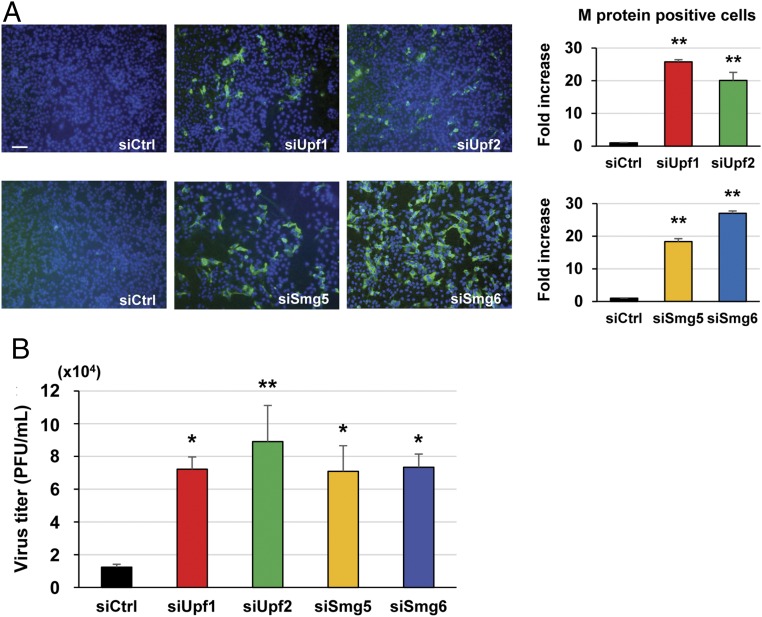
Enhancement of MHV replication from transfected genomic RNA in NMD-deficient cells. MHV genomic RNA (0.5 μg), extracted from purified MHV particles, was transfected into 17Cl-1 cells that had been treated with either control siRNA (siCtrl) or siRNAs for UPF1 (siUpf1), UPF2 (siUpf2), SMG5 (siSmg5), or SMG6 (siSmg6). (*A*) At 24 h posttransfection of the genomic RNA, the cells were stained by anti-M protein antibody (green) and DAPI (blue) (*Left*). (Scale bar: 100 μm.) The fold increase in M protein-positive cells was calculated (*Right*). (*B*) MHV titers in the culture fluid at 24 h postgenomic RNA transfection were determined by plaque assay. The data represent the mean with SEM of at least three independent experiments. Statistical analysis was done by ANOVA. *P < 0.05, **P < 0.01.

To test the hypothesis that the NMD pathway recognizes CoV mRNAs, leading to their degradation, we first examined whether the inhibition of the NMD pathway by depleting NMD factors promotes CoV replication from transfected viral genomic RNA. If CoV genomic RNA is a substrate of the NMD pathway, then depletion of NMD factors would prevent the NMD-mediated degradation of the transfected genomic RNA, resulting in efficient virus replication. In the present study, we used mouse hepatitis virus (MHV), a prototypic member of the CoV family belonging to the same genus (*Betacoronavirus*) as SARS-CoV and MERS-CoV. Treatment of 17Cl-1 cells [a mouse fibroblast cell line (18)] with specific siRNAs for different NMD factors, including UPF1, UPF2, SMG5, and SMG6, but not control siRNAs, substantially reduced the levels of the target NMD factors (*SI Appendix*, Fig. S2*A*). To examine the effect of depletion of these NMD factors on the NMD pathway, 17Cl-1 cells were transfected with an NMD reporter plasmid, NS39, or a WT reporter plasmid. Both NS39 and WT reporter plasmids encode the *Renilla* luciferase (rLuc) gene fused to the β-globin gene with or without a PTC, respectively (*SI Appendix*, Fig. S2*B*); both transcripts undergo splicing, and the NS39 reporter transcript, but not the WT reporter transcript, is a target of NMD (19). Quantitative reverse transcription–polymerase chain reaction (qRT-PCR) analysis showed a lower accumulation of NS39 reporter transcripts than WT reporter transcripts in control siRNA-transfected cells, confirming the degradation of NS39 reporter transcripts by the NMD pathway (*SI Appendix*, Fig. S2*C*). An increase in the accumulation of the NS39 reporter transcripts (relative to WT reporter transcripts) in cells depleted of UPF1, UPF2, SMG5, or SMG6 demonstrated the inhibition of the NMD pathway in these cells. We transfected these NMD-deficient cells with MHV genomic RNA and determined the number of cells positive for MHV M protein and the released virus titers at 24 h postinoculation (p.i.). The number of M protein-positive cells (Fig. 1*A*) and the virus titers (Fig. 1*B*) were both substantially higher in cells depleted of NMD factors, demonstrating that the NMD pathway inhibited virus replication from the transfected MHV genomic RNA. As UPF1 and UPF2 are core components of the NMD machinery (20, 21), we chose the depletion of these factors in 17Cl-1 cells to generate NMD-deficient cells in subsequent studies.

**Fig. 2.**
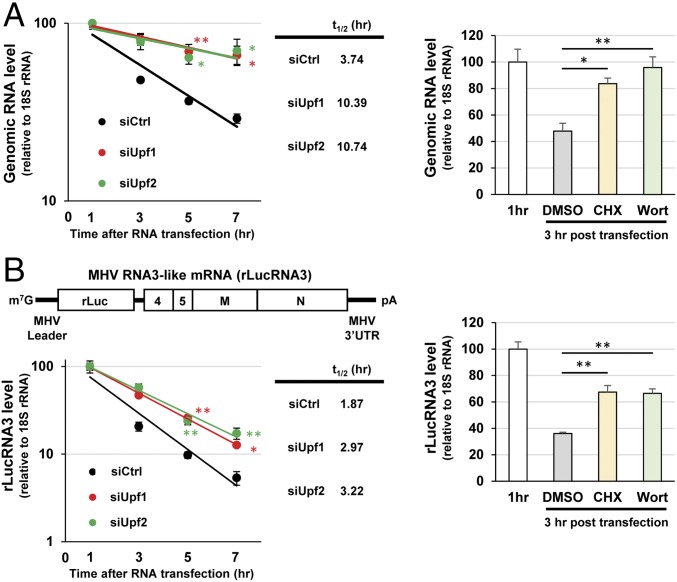
Effect of depletion of UPF1 and UPF2 on the stabilities of transfected MHV genomic RNA and subgenomiclike mRNA (rLucRNA3). (*A*, *Left*) Two micrograms per well of MHV genomic RNA was transfected into UPF1-depleted cells (siUpf1), UPF2-depleted cells (siUpf2), or cells treated with control siRNAs (siCtrl) in a 12-well plate. The levels of genomic RNA at 1, 3, 5, and 7 h posttransfection were determined by qRT-PCR and normalized to the 18S rRNA levels. (*A*, *Right*) The 17Cl-1 cells were transfected with MHV genomic RNA. After 1 h of incubation and washing to remove free genomic RNAs, total intracellular RNAs were extracted from one plate (“1 h”). The remaining plates were cultured in the presence of CHX, wortmannin (Wort), or DMSO, and the levels of genomic RNAs at 3 h posttransfection were determined by qRT-PCR and normalized to 18S rRNA levels. (*B*) Schematic diagram of subgenomic mRNA 3-like RNA (rLucRNA3) is shown on *Top*. Experiments were performed as described in *A*, except that 0.5 μg of capped and polyadenylated in vitro-synthesized rLucRNA3 was used in place of the MHV genomic RNA. The data represent the mean with SEM of at least three independent experiments. Statistical analysis was done by ANOVA. *P < 0.05, **P < 0.01. The half-life (t_1/2_) of each RNA was calculated from the slope of the trendlines.

To examine whether the transfected MHV genomic RNA is a target of the NMD pathway, we determined the degradation kinetics of the transfected genomic RNA in NMD-deficient and NMD-competent cells. Cells that had been treated with control siRNAs (NMD-competent) or with siRNAs for UPF1 or UPF2 (NMD-deficient) were transfected with MHV genomic RNA. After 1 h of incubation and washing to remove free genomic RNAs, we extracted total intracellular RNAs from the 1-h sample. To examine the kinetics of degradation of viral genomic RNA after transfection, intracellular RNAs were also extracted from NMD-deficient and NMD-competent cells at 3, 5, and 7 h posttransfection. Quantitative analyses of the genomic RNA determined by qRT-PCR showed no substantial increase in the levels of genomic RNA in the NMD-competent cells during the 7-h incubation period (Fig. 2*A*, *Left*). These data suggest that viral RNA synthesis from the transfected genomic RNA was not efficient within the first 7 h after posttransfection, which allowed us to determine the degradation kinetics of the transfected viral genomic RNA. We observed a significantly delayed degradation kinetics of the transfected genomic RNA in NMD-deficient cells compared with NMD-competent cells (Fig. 2*A*, *Left*). These data suggest that MHV genomic RNA is a substrate of the NMD pathway, which recognizes and degrades the transfected genomic RNA.

To further confirm that the transfected MHV genomic RNA was degraded by the NMD pathway, we examined the effect of NMD inhibition on the stability of the transfected genomic RNA. We used two pharmacological NMD inhibitors, cycloheximide (CHX) and wortmannin. CHX is a translation inhibitor that inhibits NMD because it is a translation-dependent event (21); wortmannin inhibits NMD by preventing SMG1-mediated UPF1 phosphorylation (22, 23), a critical step in the NMD pathway. After genomic RNA transfection, we incubated the cells with CHX, wortmannin, or dimethyl sulfoxide (DMSO), the solvent used for dissolving the inhibitors, and determined the levels of the genomic RNA at 3 h posttransfection (Fig. 2*A*, *Right*); the levels of the genomic RNA at 1 h posttransfection represented the input genomic RNA. The levels of the genomic RNA at 3 h posttransfection were significantly higher in cells treated with the NMD inhibitors, confirming that the transfected MHV genomic RNA was a target of the NMD pathway.

In addition to mRNA 1, six subgenomic mRNAs (mRNAs 2 through 7) are produced in MHV-infected cells (*SI Appendix*, Fig. S1). The subgenomic mRNAs 2 through 6 have multiple ORFs and a long 3′ UTR, both of which are NMD-inducing features in host mRNAs (6). To test whether CoV subgenomic mRNAs are also targets of the NMD pathway, we transfected an in vitro-synthesized, capped, and polyadenylated MHV subgenomic RNA 3-like reporter transcript, rLucRNA3, carrying the rLuc ORF instead of the MHV S protein ORF, and examined its stability in NMD-competent and NMD-deficient cells (Fig. 2*B*, *Left*). Similar to MHV genomic RNA, the transfected rLucRNA3 exhibited a longer half-life in NMD-deficient cells compared to NMD-competent cells (Fig. 2*B*, *Left*). Also, the levels of the transfected rLucRNA3 were higher in cells treated with NMD inhibitors (Fig. 2*B*, *Right*). These data show that the MHV subgenomic RNA 3-like reporter transcript rLucRNA3 was also a target of the NMD pathway and suggest that CoV subgenomic mRNAs can also serve as substrates of the NMD pathway.

To determine whether the targeting of MHV genomic RNA and subgenomiclike RNA for degradation by the NMD pathway is specific for these RNAs and not for any transfected RNAs, we tested the stability of a capped and polyadenylated nonviral RNA (GLA mRNA) carrying the β-globin 5′ UTR and the rLuc ORF in NMD-deficient and NMD-competent cells. The degradation kinetics of GLA mRNA was similar in both NMD-deficient and NMD-competent cells, demonstrating that GLA mRNA was not a substrate of the NMD pathway (*SI Appendix*, Fig. S3). The data demonstrate that MHV genomic RNA and rLucRNA3, but not GLA mRNA, had an NMD-inducing feature(s) that is recognized by the NMD pathway.

### Cytoplasmically Synthesized MHV Subgenomiclike RNA Is Susceptible to NMD.

**Fig. 3.**
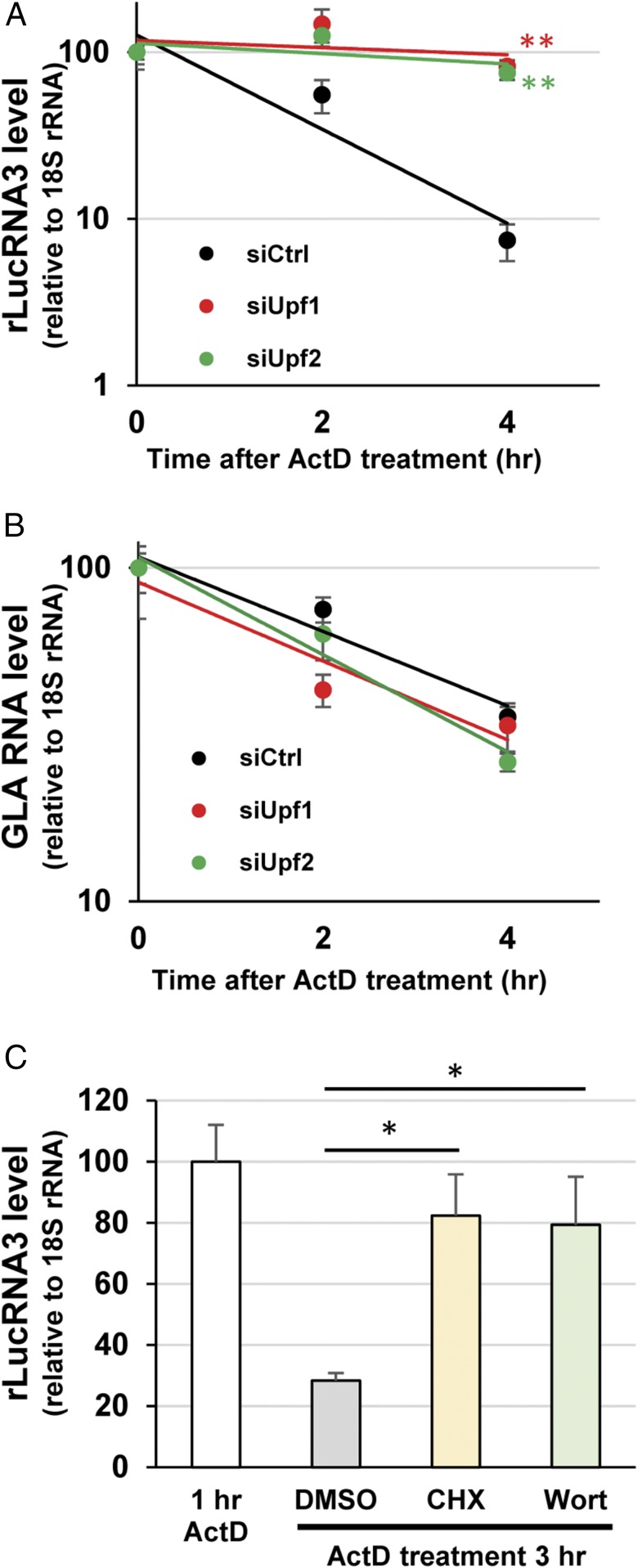
Cytoplasmically synthesized rLucRNA3, but not GLA mRNA, is susceptible to NMD. (*A*) UPF1-depleted 17Cl-1 cells (siUpf1), UPF2-depleted 17Cl-1 cells (siUpf2), or 17Cl-1 cells treated with control siRNAs (siCtrl) were cotransfected with plasmids expressing T7 polymerase, rLucRNA3, vaccinia virus capping enzyme D1R, and vaccinia virus capping enzyme D12L. At 20 h after plasmid transfection, cells were treated with actD, and levels of expressed rLucRNA3 at 0, 2, and 4 h after actD treatment were determined by qRT-PCR and normalized to 18S rRNA levels. The data represent the mean with SEM of three independent experiments. Statistical analysis was done by ANOVA. **P < 0.01. (*B*) Experiments were performed as described in *A*, except that plasmid encoding GLA RNA was used in place of that encoding rLucRNA3. (*C*) The 17Cl-1 cells were cotransfected as described in *A*. At 1 h after actD addition, DMSO, CHX, or wortmannin (Wort) was added to the cells, and levels of rLucRNA3 at 1 and 3 h after actD addition were determined by qRT-PCR and normalized to 18S rRNA levels. The data represent the mean with SEM of at least three independent experiments. Statistical analysis was done by ANOVA. *P < 0.05.

Because MHV mRNAs are synthesized in the cytoplasm of infected cells, we tested whether the NMD pathway can recognize and degrade cytoplasmically generated MHV subgenomic-like RNA (rLucRNA3). To generate capped rLucRNA3 transcripts in the cytoplasm, we cotransfected 17Cl-1 cells with four expression plasmids in the experimental group—a plasmid encoding T7 polymerase (24), two plasmids encoding the vaccinia virus capping enzymes D1R and D12L (25–28), and a rLucRNA3 plasmid carrying the rLucRNA3 sequence—downstream of a T7 promoter and upstream of a poly(A) sequence, hepatitis delta virus (HDV) ribozyme and T7 terminator. We expected that the expressed T7 RNA polymerase, along with the vaccinia virus capping enzymes, would drive the synthesis of capped and polyadenylated rLucRNA3 transcripts in the cytoplasm (29). As a control group, the plasmids expressing vaccinia virus capping enzymes were omitted, which would result in the generation of uncapped and polyadenylated rLucRNA3 transcripts. Although similar levels of rLucRNA3 transcripts were synthesized in both groups, the rLuc reporter activities were higher in the experimental group than in the control group (*SI Appendix*, Fig. S4), suggesting the generation of capped rLucRNA3 transcripts in the experimental group and resulting in the efficient cap-dependent translation of the rLuc reporter protein from the capped transcripts. To test whether the cytoplasmically generated rLucRNA3 transcripts can serve as a substrate of the NMD pathway, we examined the stability of rLucRNA3 transcripts in control siRNA-treated cells (NMD-competent) or in cells depleted of UPF1 or UPF2 (NMD-deficient). We treated the cells with actinomycin D (actD) at 20 h posttransfection to prevent new RNA synthesis, and examined the levels of preexisting rLucRNA3 transcripts at 0, 2, and 4 h after actD addition (Fig. 3*A*). We observed a significantly delayed degradation kinetics of rLucRNA3 transcripts in NMD-deficient cells compared with NMD-competent cells, suggesting that the cytoplasmically synthesized rLucRNA3 transcripts are recognized and degraded by the NMD pathway. To examine whether the targeting of cytoplasmically generated RNA transcripts by the NMD pathway is specific for capped rLucRNA3 and not for any cytoplasmically generated capped RNA, we performed similar experiments described above using a plasmid expressing GLA mRNA instead of the plasmid expressing rLucRNA3. The degradation kinetics of GLA mRNA was similar in both NMD-competent and NMD-deficient cells (Fig. 3*B*), suggesting that the NMD pathway did not affect the stability of cytoplasmically synthesized capped GLA mRNA.

To further confirm that the cytoplasmically synthesized rLucRNA3 transcripts are susceptible to NMD, we examined the effect of NMD inhibitors on the stability of capped rLucRNA3 transcripts. Cells were transfected with the four plasmids to generate capped rLucRNA3 transcripts in the cytoplasm and treated with actD, as described above. DMSO, CHX, or wortmannin was added to the cells at 1 h after actD addition, and the levels of rLucRNA3 transcripts were determined at 1 and 3 h after actD addition (Fig. 3*C*). The levels of rLucRNA3 transcripts were significantly higher in cells treated with the NMD inhibitors than in those treated with DMSO, confirming that the cytoplasmically synthesized capped rLucRNA3 transcripts are susceptible to NMD.

Together, our data strongly suggest that CoV mRNAs are a target of the NMD pathway.

### MHV Replication Induces NMD Inhibition.

**Fig. 4.**
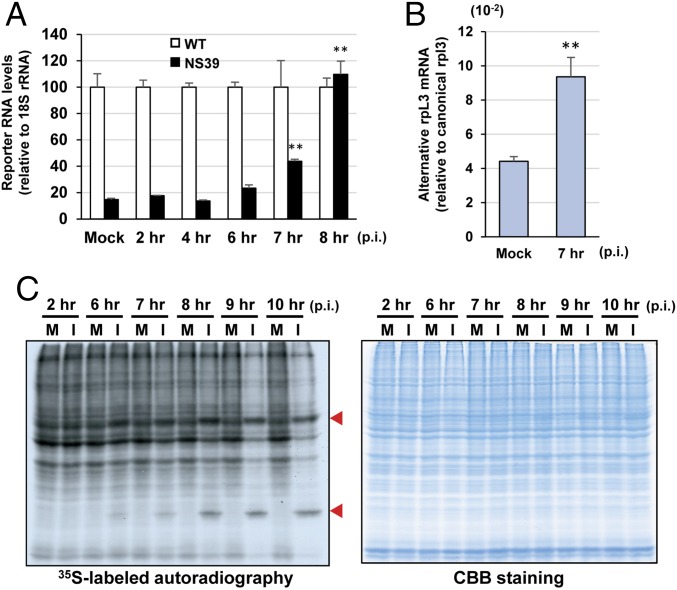
MHV replication induces NMD inhibition. (*A*) NS39 reporter cells (stably expressing NS39 reporter transcripts) and WT reporter cells (stably expressing WT reporter transcripts) were mock-infected (Mock) or infected with MHV at a multiplicity of infection (MOI) of 3. At indicated times p.i., levels of WT transcripts in WT reporter cells and NS39 reporter transcripts in NS39 reporter cells were determined by qRT-PCR and normalized to 18S rRNA levels. NS39 transcript levels are shown relative to WT (set arbitrarily at 100). (*B*) The 17Cl-1 cells were mock-infected (Mock) or infected with MHV at an MOI of 3. Total RNAs were extracted at 7 h p.i. and subjected to qRT-PCR analysis to measure the levels of alternatively spliced rpL3 mRNA relative to that of canonically spliced rpL3 mRNA. The relative ratio of the former to the latter is shown in *y* axis. The data represent the mean with SEM of at least three independent experiments. Statistical analysis was done by ANOVA. **P < 0.01. (*C*) The 17Cl-1 cells were mock-infected (M) or infected with MHV (I) at an MOI of 3. At different times p.i., cells were incubated with medium containing [^35^S]methionine/cysteine for 30 min. Cell extracts were prepared at indicated times and subjected to SDS/PAGE analysis, followed by autoradiography (*Left*) and colloidal Coomassie Brilliant Blue (CBB) staining (*Right*). Red arrowheads indicate virus-specific proteins.

The data above, demonstrating that CoV mRNAs are susceptible to NMD, led us to hypothesize that CoVs have developed a strategy to inhibit the NMD pathway in order to protect the viral mRNAs from degradation. To test this hypothesis, we generated 17Cl-1 cells stably expressing either an NMD reporter transcript (NS39, carrying a PTC) or a WT reporter transcript (lacking the PTC) by transfecting NS39 or WT reporter plasmids, respectively (19) (*SI Appendix*, Fig. S2*B*). We examined whether MHV replication protects the NS39 reporter mRNA from NMD using these stable cell lines. In the absence of MHV infection, the levels of NS39 reporter transcripts were lower than those of WT reporter transcripts due to the degradation of NS39 reporter transcripts by NMD (Mock in Fig. 4*A*). We observed a statistically significant increase in the level of NS39 reporter transcripts (relative to that of WT reporter transcripts) in MHV-infected cells at 7 h p.i. (Fig. 4*A*). At 8 h p.i., the levels of both reporter mRNAs were similar. These data reveal that MHV replication inhibited the NMD pathway starting at ∼7 h p.i.

We also tested the effect of MHV infection on the NMD pathway by examining the stability of a naturally occurring NMD target mRNA, rpL3, in MHV-infected cells. The alternatively spliced, but not the canonically spliced, rpL3 mRNA is an NMD target (30, 31). The levels of the alternatively spliced rpL3 mRNA were significantly higher in MHV-infected cells at 7 h p.i. (Fig. 4*B*), further confirming that MHV replication induced the inhibition of the NMD pathway.

As the NMD pathway is a translation-dependent event, it is indirectly suppressed due to the inhibition of translation (32–34). Because MHV replication inhibits host translation (35, 36), we tested whether the MHV-induced inhibition of NMD was primarily due to the indirect effect of host translation inhibition in MHV-infected cells. Metabolic pulse radiolabeling of MHV-infected and mock-infected cells showed that MHV-induced host translation inhibition started at ∼9 h p.i. (Fig. 4*C*), suggesting that the MHV-induced inhibition of NMD preceded the onset of host translation inhibition and was not simply an indirect effect of virus-induced translation inhibition.

### MHV N Protein Inhibits the NMD Pathway.

**Fig. 5.**
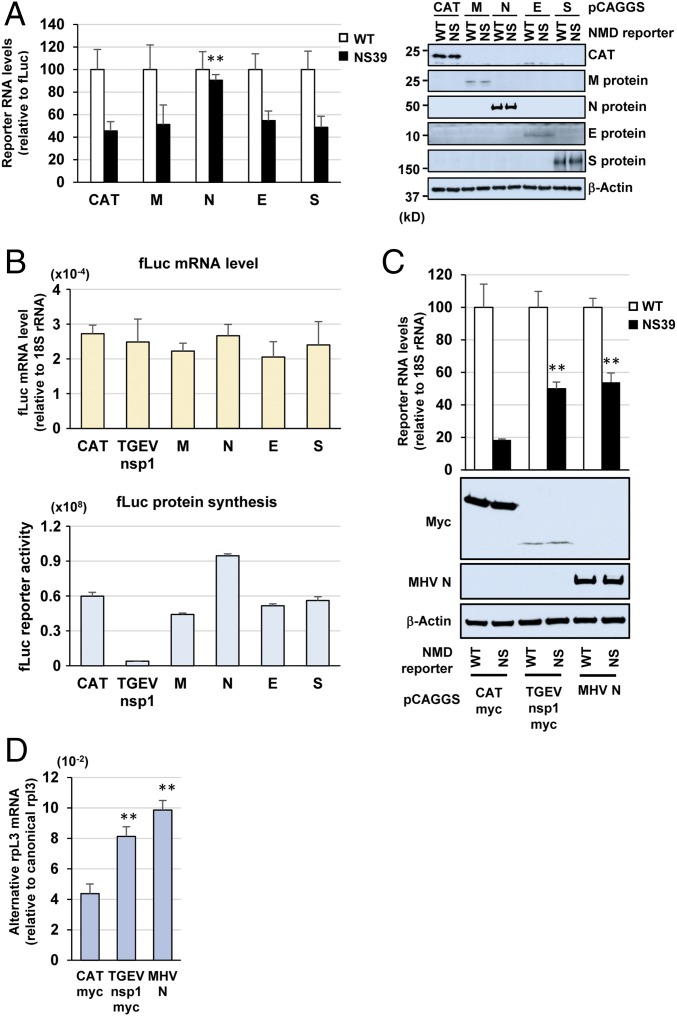
MHV N protein inhibits the NMD pathway. (*A*, *Left*) The 17Cl-1 cells were cotransfected with NS39 reporter plasmid or WT reporter plasmid along with the pCMV-fLuc plasmid expressing fLuc transcripts and plasmids encoding either CAT, M protein, N protein, E protein, or S protein. At 40 h posttransfection, the levels of WT reporter transcripts and NS39 reporter transcripts were determined by qRT-PCR and normalized to the levels of fLuc transcripts. NS39 transcript levels are shown relative to WT (set arbitrarily at 100). (*A*, *Right*) Cells were transfected as described above. Cell extracts were prepared at 40 h posttransfection and subjected to Western blot analysis to detect CAT, MHV structural proteins, and β-actin. β-Actin served as the loading control. WT, cells transfected with WT reporter plasmid; NS, cells transfected with NS39 reporter plasmid. (*B*) Cells were cotransfected as described in *A*, except that one extra sample included a plasmid expressing TGEV nsp1. Levels of fLuc transcripts (relative to 18S rRNA levels) at 40 h posttransfection (*Top*); fLuc reporter activities at 40 h posttransfection (*Bottom*). (*C*) Stable cell lines expressing either WT reporter transcripts or NS39 reporter transcripts were transfected with a plasmid expressing N protein, CATmyc, or TGEV nsp1myc. The relative levels of WT reporter transcripts and NS39 reporter transcripts are normalized to 18S rRNA at 40 h posttransfection. NS39 transcript levels are shown relative to WT (set arbitrarily at 100) (*Top*). Protein expression levels of myc-tagged CAT, myc-tagged TGEV nsp1, MHV N, and β-actin at 40 h posttransfection (*Bottom*). (*D*) The 17Cl-1 cells were transfected with a plasmid encoding CATmyc, TGEV nsp1myc, or MHV N. The levels of alternatively spliced rpL3 mRNA (relative to that of canonically spliced rpL3 mRNA) at 40 h posttransfection were determined by qRT-PCR. The data represent the mean with SEM of at least three independent experiments. Statistical analysis was done by ANOVA. **P < 0.01.

Next, we sought to identify the viral protein that exhibited an NMD inhibitory function. We hypothesized that one of the viral structural proteins associated with the incoming virions would possess an NMD inhibitory function to protect the incoming MHV genomic RNA from NMD. We established a simple assay system to determine whether one of the viral structural proteins displayed an activity to inhibit NMD. Cells were cotransfected with NS39 reporter plasmid (encoding NS39 reporter transcripts) or WT reporter plasmid (encoding WT reporter transcripts) along with a pCMV-fLuc plasmid [expressing firefly luciferase (fLuc) transcripts], and with plasmids encoding either chloramphenicol acetyltransferase (CAT) or the nonstructural protein 1 of transmissible gastroenteritis virus (TGEV nsp1), an alphaCoV; CAT and TGEV nsp1 carried a C-terminal myc tag. We hypothesized that TGEV nsp1 would inhibit the NMD pathway because it inhibits host translation without affecting the stability of host mRNAs (37)—and NMD is a translation-dependent event. At 40 h posttransfection, we examined the levels of WT and NS39 reporter transcripts relative to those of fLuc transcripts. The level of NS39 reporter transcripts was higher in TGEV nsp1-expressing cells than in CAT-expressing cells (*SI Appendix*, Fig. S5), demonstrating that TGEV nsp1 inhibited the NMD pathway. Similar experiments, using a plasmid encoding one of the MHV structural proteins (M, N, E, or S) in place of the plasmid encoding TGEV nsp1, showed that N protein expression caused a statistically significant increase in the level of NS39 reporter transcripts, demonstrating that MHV N protein inhibited the NMD pathway (Fig. 5*A*). To rule out the possibility that the inhibition of the NMD pathway by MHV N protein was due to its indirect effect on translation, we examined whether N protein expression inhibited translation by determining the levels of fLuc transcripts and fLuc reporter activities in the cells expressing the different MHV structural proteins (Fig. 5*B*). The levels of fLuc transcripts and fLuc reporter activities were similar among the samples, with no statistically significant difference, except in the case of TGEV nsp1 expression that resulted in a lower fLuc reporter activity due to its translation inhibition function. MHV N protein expression resulted in a slightly higher fLuc reporter activity. These data show that N protein inhibited the NMD pathway without suppressing translation.

To further confirm inhibition of the NMD pathway by MHV N protein, stable cell lines expressing either the NS39 or WT reporter transcripts were transfected with a plasmid expressing MHV N protein. As controls, plasmids expressing TGEV nsp1myc or CATmyc were used in place of the plasmid expressing N. The levels of NS39 reporter transcripts were higher in cells expressing MHV N or TGEV nsp1, but not CAT, suggesting that MHV N protein inhibited the NMD pathway and led to the accumulation of the NS39 reporter transcripts (Fig. 5*C*). The relative levels of the alternatively spliced rpL3 mRNA, an endogenous NMD target, were also higher in cells expressing MHV N or TGEV nsp1 (Fig. 5*D*). Together, our studies have established the role of MHV N protein as an inhibitor of the NMD pathway.

### MHV N Protein Inhibits the Degradation of Transfected Viral RNAs.

**Fig. 6.**
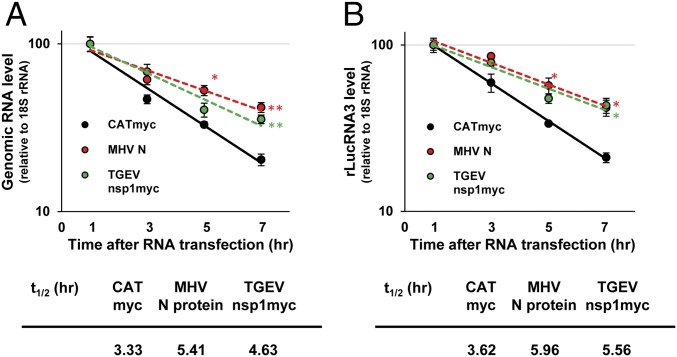
MHV N protein expression inhibits the degradation of transfected MHV genomic and subgenomiclike RNAs. In a 12-well plate, 17Cl-1 cells were transfected with a plasmid encoding MHV N, CATmyc, or TGEV nsp1myc. At 24 h posttransfection, cells were transfected with 2 μg of MHV genomic RNA (*A*) or 0.5 μg of rLucRNA3 (*B*). At 1, 3, 5, and 7 h after RNA transfection, levels of MHV genomic RNA and rLucRNA3 were determined by qRT-PCR and normalized to 18S rRNA. The data represent the mean with SEM of at least three independent experiments. Statistical analysis was done by ANOVA. *P < 0.05, **P < 0.01. The half-life (t_1/2_) of each RNA was calculated from the slope of the trendlines.

The data above suggest that N protein could protect MHV mRNAs, including the incoming viral genomic RNA and newly synthesized viral mRNAs, from NMD. To evaluate this possibility, we examined the degradation kinetics of transfected viral genomic RNA and rLucRNA3 in the presence of MHV N protein. We transfected MHV genomic RNA or the capped and polyadenylated rLucRNA3 into cells transiently expressing MHV N, myc-tagged CAT, or myc-tagged TGEV nsp1 and determined the levels of the transfected RNAs at various times posttransfection (Fig. 6). We observed a delayed degradation kinetics with longer half-lives for the RNAs in cells expressing MHV N or TGEV nsp1. These data are consistent with the notion that MHV N protein inhibits the degradation of viral mRNAs by NMD.

### NMD Pathway Inhibits Optimal MHV Replication by Targeting Viral mRNAs Synthesized Early in Infection.

**Fig. 7.**
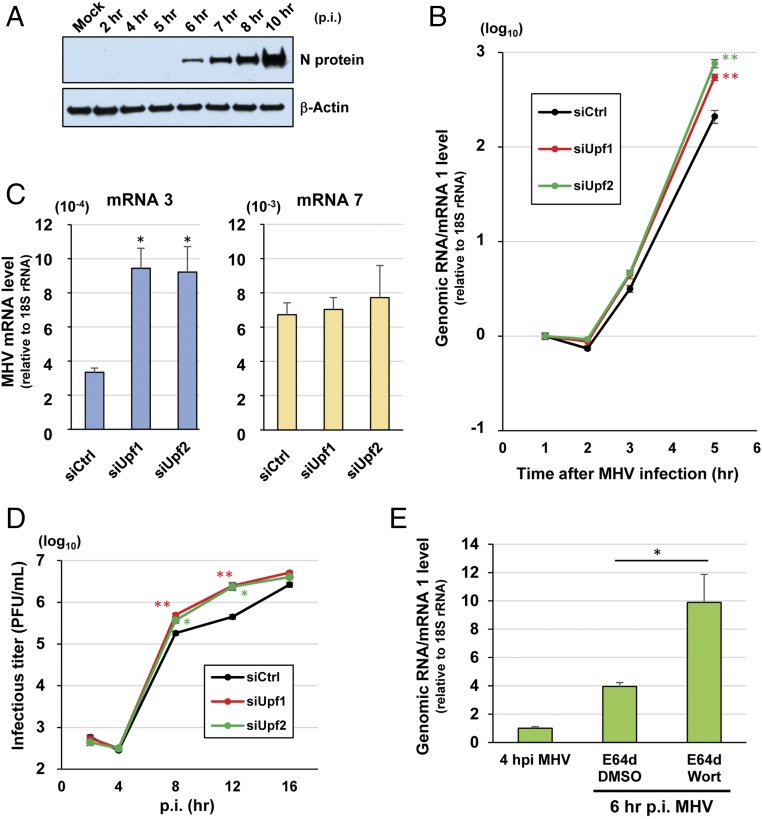
NMD inhibition promotes the accumulation of MHV mRNAs early in infection, facilitating virus replication. (*A*) The 17Cl-1 cells were mock-infected (Mock) or infected with MHV at a multiplicity of infection (MOI) of 0.1. Accumulation of N protein, at the indicated times p.i., was examined by Western blot analysis. β-Actin served as the loading control. (*B*) UPF1-depleted 17Cl-1 cells (siUpf1), UPF2-depleted 17Cl-1 cells (siUpf2), or control siRNA-treated 17Cl-1 cells (siCtrl) were infected with MHV at an MOI of 0.1. The levels of genomic RNA/mRNA 1 (relative to 18S rRNA) at 1, 2, 3, and 5 h p.i. were determined by qRT-PCR. (*C*) Cells were infected with MHV as described in *B*. The levels of mRNA 3 (*Left*) and mRNA 7 (*Right*) (relative to 18S rRNA) at 5 h p.i. were determined by qRT-PCR. Because the levels of these mRNAs at 1, 2, or 3 h p.i. were below the limit of detection, we could not accurately determine their relative levels at these early time points. (*D*) siRNA-treated cells were infected with MHV as described in *B*. Virus titers in culture supernatant at 2, 4, 8, 12, and 16 h p.i. are shown. (*E*) The 17Cl-1 cells were infected with MHV at an MOI of 0.1. Cells were treated with 300 μg/mL E64d and either 20 μM wortmannin (Wort) or DMSO at 4 h p.i. The levels of mRNA 1 (relative to 18S rRNA) at 4 and 6 h p.i. were determined by qRT-PCR. The data represent the mean with SEM of at least three independent experiments. Statistical analysis was done by ANOVA. *P < 0.05, **P < 0.01.

Although N protein is a component of the helical nucleocapsid in the incoming virion that could protect the incoming genomic RNA from NMD, there is a possibility that the newly synthesized viral mRNAs early in infection are subject to NMD due to low levels of accumulation of N protein. Consistent with this notion, the accumulation of MHV N protein, which is translated from viral mRNA 7 (*SI Appendix*, Fig. S1) early in infection, was low, with detectable levels starting only at 6 h p.i. (Fig. 7*A*). To test the possibility that MHV mRNAs synthesized early in infection are susceptible to NMD, we inoculated MHV into cells treated with control siRNAs (NMD-competent) or with siRNAs for UPF1 or UPF2 (NMD-deficient) and examined the levels of viral mRNAs at different times p.i. (Fig. 7 *B* and *C*). The levels of incoming genomic RNA in NMD-competent and NMD-deficient cells were not significantly different during the first 2 h p.i. (Fig. 7*B*). The level of newly synthesized mRNA 1, which was initially detected at 3 h p.i., was significantly higher in NMD-deficient cells compared to NMD-competent cells at 5 h p.i. (Fig. 7*B*). The level of mRNA 3, but not mRNA 7, was also higher in NMD-deficient cells than in NMD-competent cells at 5 h p.i. (Fig. 7*C*). At 5 h p.i., the difference in the levels of mRNA 1 between NMD-deficient cells and NMD-competent cells was more pronounced than the difference in the levels of mRNA 3 among these cells. These data suggest that early in infection, MHV mRNAs 1 and 3, but not the smallest viral mRNA (mRNA 7), were subjected to NMD. These data also revealed that the inhibition of the NMD pathway by the depletion of NMD factors promoted the accumulation of viral mRNAs, especially the longer mRNAs, early in infection.

To determine whether the higher levels of accumulation of viral mRNAs in NMD-deficient cells had an impact on the production of infectious MHV particles, we examined the replication kinetics of MHV in control siRNA-treated cells (NMD-competent) or in cells treated with siRNAs for UPF1 or UPF2 (NMD-deficient) (Fig. 7*D*). Higher titers of MHV were released from NMD-deficient cells, starting from 8 to 12 h p.i., indicating that the accumulation of higher amounts of viral mRNAs early in infection in NMD-deficient cells facilitated the production of higher titers of MHV.

To gain further evidence that newly synthesized MHV mRNA 1 is susceptible to NMD early in infection, we examined the effect of NMD inhibition on the stability of mRNA 1 (which is synthesized early in infection) in cells treated with E64d, a specific, irreversible inhibitor of cysteine proteinases that inhibits the processing of viral gene 1 polyprotein, resulting in the inhibition of CoV RNA synthesis (38). Treatment with E64d causes a reduction in the rate of RNA synthesis, which allowed us to measure the NMD-mediated turnover of newly synthesized mRNA 1 by comparing its levels in MHV-infected cells treated with the NMD inhibitor wortmannin or DMSO at 6 h p.i. First, we tested the effect of E64d treatment on MHV RNA synthesis and found that it efficiently inhibited, albeit incompletely, the synthesis of mRNA 1 (*SI Appendix*, Fig. S6*A*). Furthermore, E64d-mediated inhibition of viral mRNA synthesis also severely inhibited the accumulation of MHV N protein (*SI Appendix*, Fig. S6*B*), excluding the effect of its role as an NMD inhibitor in our experimental approach. Importantly, E64d treatment did not inhibit the NMD pathway (*SI Appendix*, Fig. S6*C*). These data show the feasibility of using E64d in our experiment to inhibit viral mRNA synthesis. We treated MHV-infected cells with E64d and wortmannin or with E64d and DMSO from 4 to 6 h p.i., and measured the levels of mRNA 1 at 4 and 6 h p.i. (Fig. 7*E*). The level of mRNA 1 was significantly higher in E64d-treated cells incubated with wortmannin than in those incubated with DMSO. To exclude the possibility that wortmannin treatment promoted viral mRNA synthesis, leading to higher levels of accumulation of mRNA 1, we examined the effect of wortmannin treatment on mRNA 1 accumulation in the absence of E64d. The level of mRNA 1 was slightly lower, although not statistically significantly, in wortmannin-treated cells than in DMSO-treated cells (*SI Appendix*, Fig. S6*D*), strongly suggesting that wortmannin treatment did not facilitate viral mRNA synthesis. Our data suggest that NMD inhibition by wortmannin in E64d-treated cells protects the newly synthesized mRNA 1 from degradation, resulting in its higher level of accumulation.

Our data show that NMD inhibition, either by depletion of the NMD factors UPF1 and UPF2 or by wortmannin treatment, resulted in higher levels of MHV mRNA 1 accumulation, which strongly suggests that newly synthesized MHV mRNA 1 is susceptible to NMD early in infection.

## Discussion

The present study investigated the interplay between CoVs and the NMD pathway by using MHV as a prototypic CoV. We show that inhibition of the NMD pathway, by the depletion of NMD factors, significantly delayed the degradation of the transfected MHV genomic RNA, thereby facilitating efficient virus replication (Fig. 1). Depletion of NMD factors also delayed the rapid degradation of transfected rLucRNA3, a viral subgenomiclike mRNA (Fig. 2), as well as cytoplasmically synthesized rLucRNA3 (Fig. 3). Pharmacological inhibition of the NMD pathway, using CHX and wortmannin, also inhibited the decay of transfected genomic RNA and rLucRNA3 (Fig. 2) as well as cytoplasmically synthesized rLucRNA3 (Fig. 3), establishing that MHV mRNAs are targets of the NMD pathway. We also reveal that MHV replication inhibited the NMD pathway before the onset of virus-induced translation inhibition (Fig. 4). It should be noted that efficient viral protein synthesis continued even after the induction of host protein synthesis inhibition (Fig. 4*C*). Because the actively translating viral mRNAs would be susceptible to NMD, our data suggest that MHV-induced inhibition of the NMD pathway is important for efficient viral gene expression. We identified MHV N protein as an inhibitor of the NMD pathway (Fig. 5). However, our data did not exclude the possibility of other MHV proteins (including gene 1 or accessory proteins) possessing the activity to inhibit the NMD pathway. Although MHV N protein also protected transfected viral genomic RNA and rLucRNA3 from rapid degradation (Fig. 6), providing indirect evidence for its role in protecting viral mRNAs from NMD in infected cells, our data do not rule out the possibility that N protein protected the viral mRNAs from other RNA decay pathways. Subsequently, our studies revealed that early in infection, before the efficient accumulation of N protein, newly synthesized MHV mRNAs 1 and 3 are susceptible to NMD and that inhibition of the NMD pathway by the depletion of NMD factors promoted the accumulation of viral mRNAs early in infection, leading to the production of higher titers of MHV (Fig. 7). Together, the present study’s results strongly suggest that the NMD pathway recognizes and targets MHV mRNAs for degradation and that the inhibition of the NMD pathway, mediated by MHV N protein, is important for efficient virus replication.

Past studies have revealed various types of interactions between viruses and the NMD pathway (39). Genomic-wide siRNA screening for host factors involved in the replication of Semliki forest virus (SFV), an alphavirus, in mammalian cells (16) and a genetic screen in *Arabidopsis* for factors involved in the replication of Potato virus X (PVX), a positive-strand RNA virus in plants (13), led to the discovery of the role of NMD factors in the replication of these cytoplasmic RNA viruses. Like CoVs, these viruses carry a single-stranded, positive-sense RNA genome with a long 3′ UTR and produce one or more subgenomic mRNAs that form a nested-set structure in infected cells. In the case of retroviruses, the viral mRNAs are synthesized in the nucleus and many of them undergo splicing; therefore, it is not surprising that retroviruses have evolved to control the NMD pathway. Rous sarcoma virus, an avian retrovirus, carries an RNA stability element that facilitates the escape of full-length viral RNA (carrying a long 3′ UTR) from degradation by the NMD pathway (40). Human T-lymphotropic virus type I encodes the NMD inhibitors Tax and Rex, which prolong the half-life of viral mRNAs (41, 42), whereas the NMD factor UPF1 serves as a positive regulator of HIV type 1 replication (43–45).

In principle, the NMD pathway in mammalian cells acts on newly synthesized mRNAs that undergo splicing and harbor an EJC (21, 46). Our data showing that MHV mRNAs of cytoplasmic origin, which do not undergo splicing and lack EJC, are also targeted by the NMD pathway suggest that the NMD pathway is able to recognize and target mRNAs that lack canonical NMD-inducing features. Nonetheless, the recognition and targeting of mRNAs of cytoplasmic origin by the NMD pathway followed some fundamental principles that govern the turnover of mammalian mRNAs by NMD, including the requirement of NMD factors UPF1, UPF2, SMG5, and SMG6; UPF1 phosphorylation; and the translation of target mRNAs (Figs. 1–3). However, it is unclear whether other NMD factors that play a critical role in the NMD of mammalian mRNAs are also required for NMD-mediated turnover of CoV mRNAs. Further studies are warranted to identify the host factors that are involved in the targeting of cytoplasmically synthesized viral mRNAs by the NMD pathway.

In addition to the well-characterized role of the NMD pathway as a quality control system for eliminating aberrant host mRNAs, present and past studies (13, 16) have highlighted a biological role for the NMD pathway as an intrinsic host defense mechanism against cytoplasmic RNA viruses. It is conceivable that many cytoplasmic RNA viruses have evolved to evade the NMD pathway altogether by eliminating NMD-inducing RNA features from the viral mRNAs. One of the NMD-inducing features in mammalian mRNAs is the presence of a long 3′ UTR (10, 13, 14). It has been demonstrated that the long 3′ UTR of subgenomic RNA of PVX is targeted by the NMD pathway (13), but shortening the length of the 3′ UTR in alphavirus genomic RNA did not protect the RNA from NMD (16), suggesting the presence of one or more additional NMD-inducing features in the alphavirus genomic RNA. We observed that the transfected or cytoplasmically synthesized rLucRNA3, but not GLA mRNA, was susceptible to NMD, suggesting that the long 3′ UTR is one of the NMD-activating features in rLucRNA3 that triggered its NMD-mediated degradation (Figs. 2 and 3). Furthermore, the inhibition of the NMD pathway promoted the accumulation of viral mRNAs early in infection, with the effect being more pronounced for longer viral mRNAs (Fig. 7). These data are also consistent with the long 3′ UTR being one of the NMD-activating features in susceptible mRNAs (10, 13, 14), which implies that the long 3′ UTR in cytoplasmically synthesized mRNAs, including CoV mRNAs, is a trigger for NMD. Because the cytoplasmically generated rLucRNA3 transcripts were susceptible to NMD (Fig. 3), these transcripts can be used to delineate the NMD-inducing RNA features in CoV mRNAs.

A possible mechanism employed by cytoplasmic RNA viruses to subvert the NMD pathway is through one or more viral proteins that inhibit the NMD pathway or by altering the cellular environment, rendering it unfavorable for the NMD pathway. In alphavirus, the SFV mutant lacking the C-terminal part of nsp3 was more susceptible to UPF1-mediated degradation (16), although it is unclear whether nsp3 has an NMD inhibitory function. The core protein of hepatitis C virus (HCV) binds to an EJC recycling factor and prevents the factor from interacting with other components of the EJC, leading to the inhibition of the NMD pathway in infected cells (17). Unlike in the case of MHV genomic RNA, UPF1 depletion does not affect the stability of transfected HCV genomic RNA (47). However, these data do not rule out the possibility of HCV genomic RNA as a target of NMD, because the core protein, synthesized from the transfected HCV genomic RNA, could have protected the HCV genomic RNA from NMD. Currently, the biological significance of NMD inhibition in HCV replication is unclear. Our present study shows that MHV replication inhibited the NMD pathway (Fig. 4) and reveals the role of MHV N protein as an NMD inhibitor (Fig. 5) that protects transfected viral mRNA or viruslike mRNA from rapid degradation (Fig. 6). Our data also strongly suggest that the inhibition of the NMD pathway is important for the protection of viral mRNAs from rapid decay, thereby facilitating efficient virus replication (Fig. 7). Hence, our study highlights the biological importance of the NMD pathway in controlling the stability of mRNAs of a cytoplasmic RNA virus. Our study also directly demonstrates the recognition and targeting of mRNAs of a cytoplasmic RNA virus by the NMD pathway, highlighting the importance of virus-mediated subversion of the NMD pathway for efficient replication of a cytoplasmic RNA virus.

In addition to our main finding that MHV mRNAs are vulnerable to the NMD pathway, we also reveal the role of MHV N protein as an inhibitor of the NMD pathway. It has been shown that in CoV reverse genetics systems, the addition of transcripts encoding N protein to in vitro-synthesized full-length CoV transcripts for electroporation enhances the recovery of infectious viruses (48–51). It is possible that the N protein, translated from the transcripts, plays a role in protecting the full-length CoV transcripts from NMD, facilitating the efficient recovery of CoVs. As the mRNAs of all CoVs carry the NMD-activating features (i.e., the presence of multiple ORFs and a long 3′ UTR), it is highly likely that the NMD inhibition function is a common biological property of CoV N protein. Because the genomes of all members of the order Nidovirales, including CoVs and arteriviruses, share these NMD-activating features, it is conceivable to speculate that in addition to MHV, the mRNAs of other nidoviruses are also a target of the NMD pathway and that nidoviruses have evolved to encode a viral protein that inhibits the NMD pathway. Analyses of the cellular interactome of the CoV infectious bronchitis virus N protein (52) and that of the arterivirus porcine reproductive and respiratory syndrome virus N protein (53) suggested an interaction of these N proteins with UPF1. However, the biological significance of these putative interactions has not been examined. It is tempting to speculate that CoV N protein binds and sequesters UPF1 away from the NMD machinery, resulting in the inhibition of the NMD pathway. It would be valuable and significant to clarify the mechanism of N protein-mediated NMD inhibition, as disrupting the NMD inhibitory function of N protein would represent an avenue for controlling CoV replication.

Although our data suggest that the inhibition of the NMD pathway by N protein could serve to protect viral mRNAs from NMD, thereby facilitating efficient viral gene expression and replication, the N protein-mediated inhibition of the NMD pathway in CoV-infected cells could also have an impact on other cellular responses to viral infections. There is accumulating evidence suggesting an active role for the NMD pathway in the regulation of immune responses and the inhibition of the NMD pathway under various cellular stress conditions, including infection (20). This raises the possibility that the inhibition of host NMD activity by CoV N protein could negatively affect the regulation of stress and immune responses by the NMD pathway in CoV-infected animals, contributing to the pathogenicity of CoVs.

## Materials and Methods

### Cells and Viruses.

The 17Cl-1 cells were cultured in Dulbecco’s modified Eagle’s medium (DMEM) containing 10% FBS, and the mouse astrocytoma cell line DBT cells (54) were cultured in Eagle’s MEM containing 10% newborn calf serum and 10% tryptose phosphate broth. The plaque-cloned A59 strain of MHV was propagated and titrated in DBT cells.

### Plasmids.

The two NMD reporter plasmids, NS39 and WT, and pCMV-fLuc were described previously (19). The eukaryotic expression plasmid, pCAGGS, was used to express CAT, myc-tagged CAT, TGEV nsp1, myc-tagged TGEV nsp1, MHV M protein, MHV N protein, MHV E protein, and MHV S protein. The T7-rLucRNA3 plasmid carried the entire MHV mRNA 3 region minus the S gene, which was replaced by the rLuc gene, downstream of a T7 promoter and upstream of a poly(A) sequence, HDV ribozyme and T7 terminator. The T7-GLA plasmid carried the β-globin 5′ UTR and the rLuc gene (55) downstream of the T7 promoter and upstream of the poly(A) sequence, HDV ribozyme and T7 terminator. The plasmids were transfected into 17Cl-1 cells using TransIT-LT1 reagent (Mirus) or Lipofectamine 2000 (Thermo Fisher Scientific).

### Isolation of MHV Genomic RNA.

MHV was purified by using sucrose gradient centrifugation as described previously (56). Genomic RNA was extracted from the pelleted virion using TRIzol reagent (Thermo Fisher Scientific) and purified by phenol–chloroform and isopropanol precipitation.

### siRNA Transfection.

ON-TARGET plus SMART pool for each target mRNA and ON-TARGET plus nontargeting pool, a negative control siRNA, were purchased from Dharmacon. The 17Cl-1 cells were plated in six-well plates in DMEM supplemented with 10% FBS without antibiotics. After 24 h of incubation, cells at 50 to 60% of confluence were transfected with 25 nM siRNA using TransIT-siQUEST transfection reagent (Mirus) according to the manufacturer’s instructions. After 24 h of incubation, cells were plated onto 12-well plates and further incubated for 24 h before RNA transfection or virus infection.

### RNA Transfection.

Capped and polyadenylated T7 rLucRNA3 and GLA mRNA transcripts were synthesized in vitro by mMESSAGE mMACHINE kit (Thermo Fisher Scientific) using linearized pSV40T7-rLucRNA3 and pSV40T7-GLA plasmids as templates. The in vitro-synthesized mRNAs or MHV genomic RNA was transfected into siRNA-treated cells using the TransIT-mRNA Transfection Kit (Mirus) according to the manufacturer’s instruction and incubated for 1 h. After removal of the inoculum, the cells were washed twice with PBS and incubated with fresh complete media. At different times after RNA transfection, cells were directly lysed with TRIzol reagent, followed by RNA isolation. For testing MHV replication, culture fluid was collected at 24 h posttransfection. For immunofluorescence microscopy analysis, cells in chamber slides were fixed at 24 h posttransfection.

### Immunofluorescence Analysis.

Cultured cells were washed once with PBS and air-dried for 5 min at room temperature. Next, cells were fixed with methanol–acetone (1:1) for 15 min at room temperature, air-dried for 5 min, and stored at −20 °C until use. The cells were washed once with PBS and incubated with anti-MHV-M (J2.7) antibody diluted in PBS containing 3% BSA for 3 h at 37 °C in a moist chamber. After washing three times with PBS, the cells were incubated with Alexa 488-conjugated anti-mouse IgG (Thermo Fisher Scientific) in PBS containing 3% BSA for 1 h at room temperature. After incubation, the cells were washed three times with PBS and mounted in VECTASHIELD mounting medium with DAPI (Vector Laboratories). The samples were observed using an AxioPhot 2 epifluorescence microscope.

### Generation of RNA Transcripts in the Cytoplasm.

The 17Cl-1 cells were cotransfected with plasmids encoding either T7-rLucRNA3 plasmid or T7-GLA plasmid, along with plasmids encoding T7 polymerase, T7opt (24), vaccinia virus capping enzyme D1R (25) (89160; Addgene), or vaccinia virus capping enzyme D12L (25) (89161; Addgene). For the generation of uncapped transcripts, the plasmids encoding vaccinia virus capping enzymes were omitted and pCAGGS-CAT was used to adjust the total amount of plasmids. At 20 h posttransfection, the cells were treated with 4 μg/mL actD. In some experiments, 100 μg/mL CHX (Sigma) or 20 μM wortmannin (Sigma) was added to cells at 1 h after actD addition.

### Generation of Stable Cell Lines Expressing NMD Reporter RNAs.

In a six-well plate, 17Cl-1 cells were transfected with either 1.0 μg of WT reporter plasmid or NS39 reporter plasmid using Lipofectamine 2000 according to the manufacturer’s instructions. After 24 h of incubation, the cells were trypsinized and plated onto a 10-cm dish with DMEM containing 10% FBS and 400 μg/mL G418. After two to three passages with G418 for selection, the bulk-populated stable reporter cells were used for experiments.

### RNA Isolation and qRT-PCR.

For qRT-PCR analyses, we isolated total RNA from naïve 17Cl-1 cells or from cells transfected with appropriate plasmids using TRIzol reagent and the Direct-zol RNA Miniprep Kit (Zymo Research). One microgram of total RNA was reverse transcribed by SuperScript III enzyme (Thermo Fisher Scientific) for cDNA synthesis. The expression levels of target genes were determined by CFX96 Touch Real-Time PCR Detection System and SsoAdvanced Universal SYBR Green Supermix (Bio-Rad). The primers for qPCR are shown in *SI Appendix*, Table S1.

### Western Blot Analysis.

Whole-cell lysates were electrophoresed on 4 to 20% or 7.5% polyacrylamide gels and transferred to polyvinylidene difluoride membrane (Bio-Rad). The membrane was probed with specific antibodies for UPF1 (9435; Cell Signaling Technology), UPF2 (sc-20227; Santa Cruz Biotechnology), SMG5 (ab33033; Abcam), SMG6 (ab87539; Abcam), myc (4A6 clone, 05-724; MilliporeSigma), CAT (ab50151; Abcam), and actin (sc-1616; Santa Cruz Biotechnology). The α-M protein J1.3 and α-N protein J3.3 antibodies from John Fleming, University of Wisconsin–Madison, Madison, WI, the α-MHV-JHM antibody for S protein detection from Susan Baker, Loyola University Stritch School of Medicine, Maywood, IL, and the α-E protein 5b antibody from Julian Leibowitz, Texas A&M University, College Station, TX were used to detect MHV proteins. The specific bands were detected by ECL Western Blotting Detection Reagent.

### Radiolabeling of Cells.

MHV-infected 17Cl-1 cells were incubated in methionine-free medium for 30 min and then incubated in medium containing 50 μCi/mL [^35^S]methionine/cysteine (1,000 Ci/mmol; MP Biomedicals) for 30 min. Whole-cell lysates were prepared in SDS sample buffer and analyzed on 12.5% SDS/PAGE.

### Statistical Analysis.

Analysis of variance (ANOVA) with the Tukey test-considered significant difference as *P* < 0.05 was used for MHV infectious titer, cell numbers, gene expression, and RNA degradation experiments (GraphPad Prism 4).

## Supplementary Material

Supplementary File
