# Supplementary material for: Interplay between coronavirus, a cytoplasmic RNA virus, and nonsense-mediated mRNA decay pathway
Source: Proc Natl Acad Sci U S A. 2018 Oct 8;115(43):E10157–66. doi: 10.1073/pnas.1811675115 (PMC6205489; doi:10.1073/pnas.1811675115)
Supplement: Supplementary File [file pnas.1811675115.sapp.pdf]

PNAS
www.pnas.org

Supplementary Information for

Interplay between coronavirus, a cytoplasmic RNA virus, and nonsense-mediated mRNA decay pathway

Masami Wada, Kumari G. Lokugamage, Keisuke Nakagawa, Krishna Narayanan and Shinji Makino

Shinji Makino
Email: shmakino@utmb.edu

This PDF file includes:

Supplementary text
Figs. S1 to S6
Table S1

www.pnas.org/cgi/doi/10.1073/pnas.1811675115

Supplementary Materials and Methods

Genomic RNA transfection and MHV infection in NMD-deficient cells. The negative control siRNA (ON-TARGET plus non-targeting pool) and the specific siRNAs (ON-TARGET plus SMART pool) for the knockdown of key NMD effectors, Upf1, Upf2, Smg5 and Smg6, were purchased from Dharmacon. 17Cl-1 cells were plated in 6-well plates in Dulbecco's modified Eagle's medium (DMEM) supplemented with 10% (v/v) fetal bovine serum (FBS) without antibiotics, 24 h before siRNA transfection. At 50-60% confluency, cells were transfected with siRNA using TransIT-siQUEST transfection reagent (Mirus) according to the manufacturer's instructions and incubated at 37□ in a CO_2 incubator. After incubating for 24 h, the siRNA-treated cells were split and plated onto 12-well plates for virus collection or 4-well chamber slides for IFA. After incubation at 37□ in a CO_2 incubator for 24 h, cells were transfected with MHV genomic RNA (1.5 μg/well) using TransIT mRNA reagent (Mirus) or infected with MHV. The supernatant medium was collected from MHV genomic RNA transfected /MHV infected cells or the cells in chamber slides were fixed at 24 h post-transfection for virus titration and IFA, respectively. The knockdown efficiency of siRNAs was confirmed by Western blot.

RNA synthesis by *in vitro* transcription. pSV40T7-GLA and pSV40T7-rLucRNA3 were linearized with PmeI and XbaI, respectively. After digestion, the templates were purified by phenol/chloroform and ethanol precipitation. All RNAs were transcribed by using mMESSAGE mMACHINE T7 transcription kit.

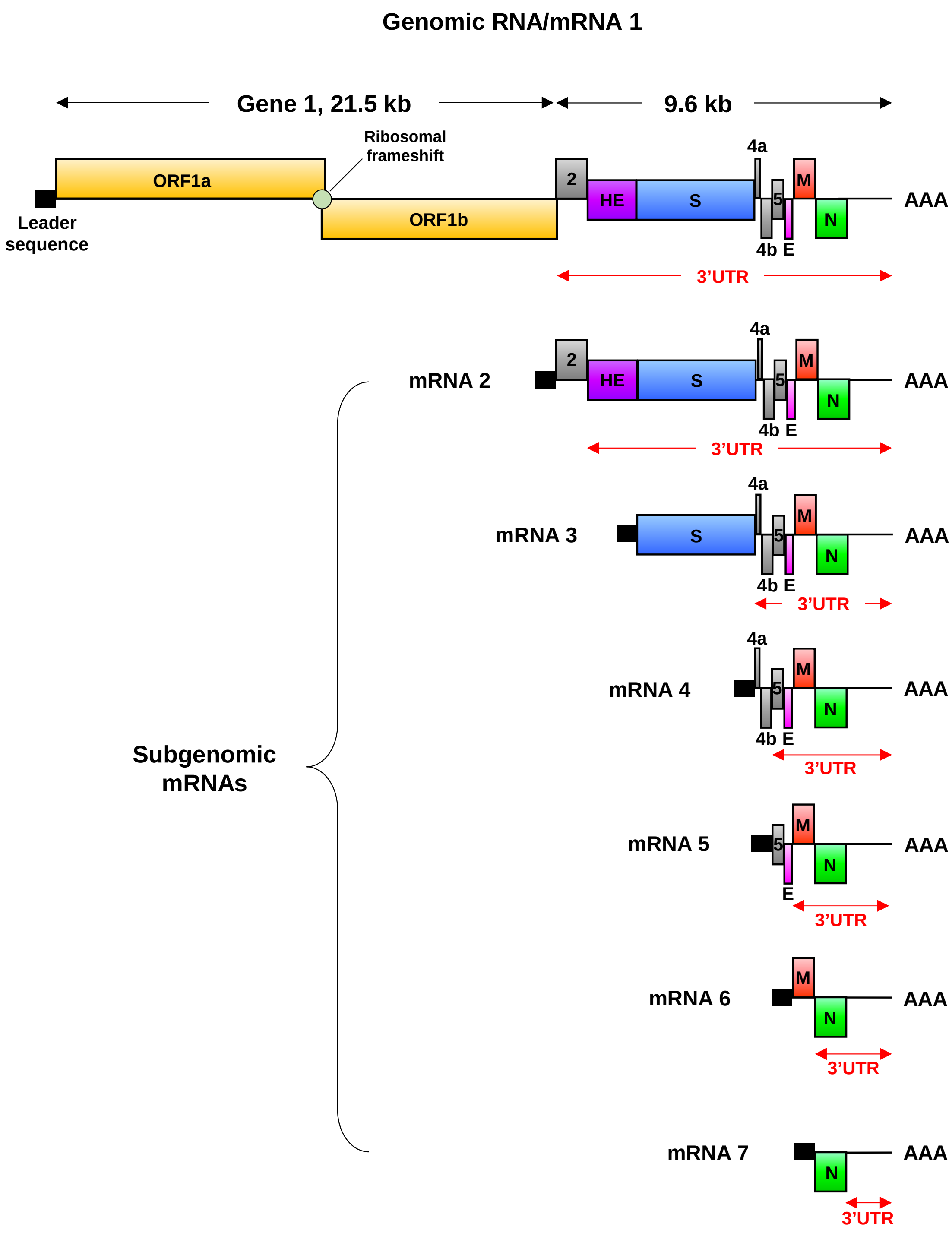

Fig. S1. Schematic diagrams of MHV genomic RNA/mRNA 1 and subgenomic mRNAs. The 5' and 3' untranslated regions (UTRs) and viral open reading frames (boxes) are not drawn according to scale. Note that all the viral mRNAs, except for mRNA 7, have multiple ORFs and in principle, only the most 5' ORF of each mRNA is used for translation. The 3' UTR of each mRNA is indicated by a red arrow.

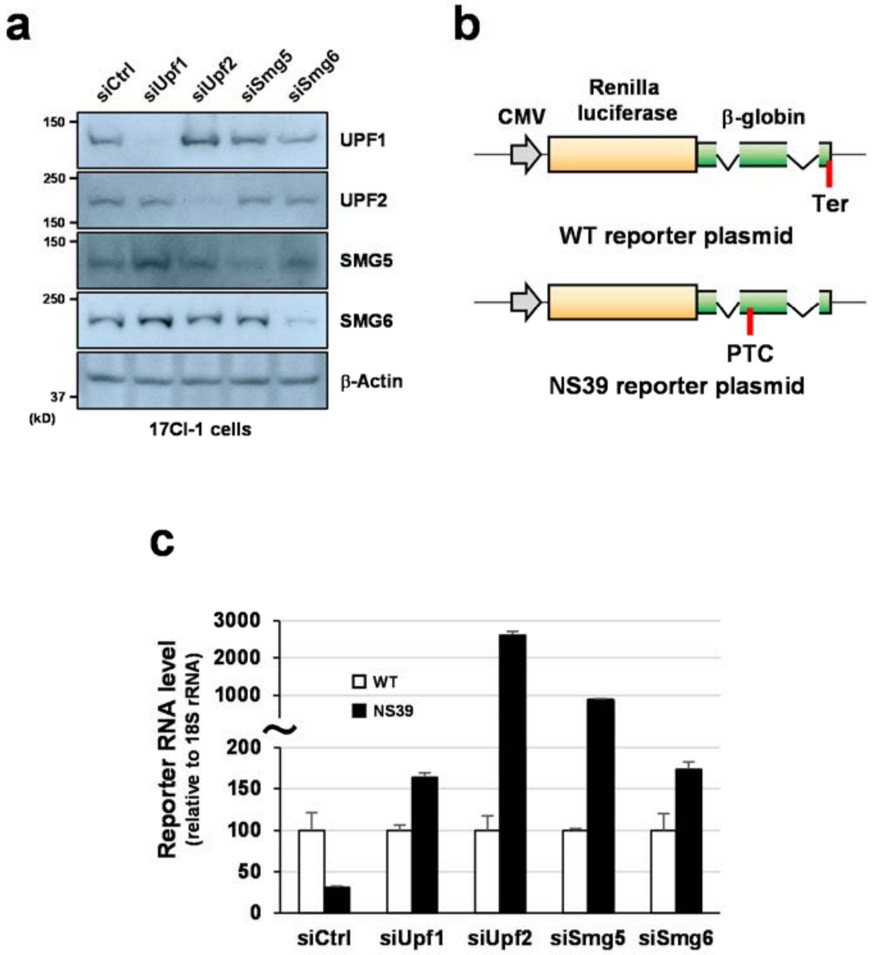

Fig. S2. siRNA-mediated depletion of NMD factors in 17Cl-1 cells. (a) 17Cl-1 cells were transfected with control siRNA (siCtrl), siRNAs for UPF1 (siUpf1), UPF2 (siUpf2), SMG5 (siSmg5) or SMG6 (siSmg6). The abundance of UPF1, UPF2, SMG5, SMG6 and β-actin at 48 h post-transfection was examined by Western blot analysis. (b) Schematic diagrams of WT reporter plasmid, encoding WT transcripts lacking the PTC (top), and NMD reporter plasmid, encoding NS39 transcripts with PTC (bottom). CMV, CMV promoter; Ter, termination codon; PTC, premature termination codon. (c) 17Cl-1 cells, treated with either control siRNA or siRNAs for UPF1, UPF2, SMG5 or SMG6, were transfected with 0.05 μg of WT reporter plasmid or NS39 reporter plasmid. At 24 h post-transfection, the levels of WT and NS39 reporter transcripts were determined by RT-qPCR and normalized to 18S rRNA levels. The NS39 transcript levels are shown relative to WT (set arbitrarily at 100).

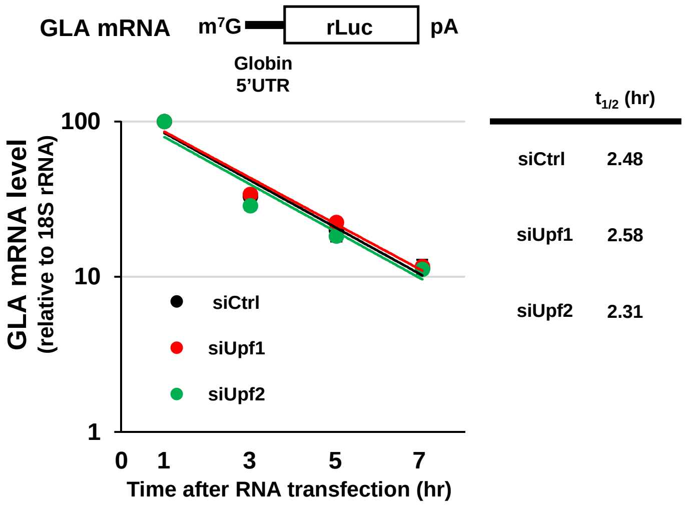

Fig. S3. Effect of depletion of UPF1 and UPF2 on the stability of transfected nonviral RNA. Schematic diagram of a nonviral RNA, GLA mRNA, is shown on top. 0.3 µg of capped and polyadenylated in vitro-synthesized GLA mRNA was transfected into UPF1-depleted cells (siUpf1), UPF2-depleted cells (siUpf2) or control siRNA-treated cells (siCtrl) in a 12-well plate. The levels of GLA mRNA at 1, 3, 5 and 7 h post-transfection were determined by RT-qPCR and normalized to18S rRNA levels. The half-life ($t_{1/2}$) of GLA mRNA was calculated from the slope of the trendlines.

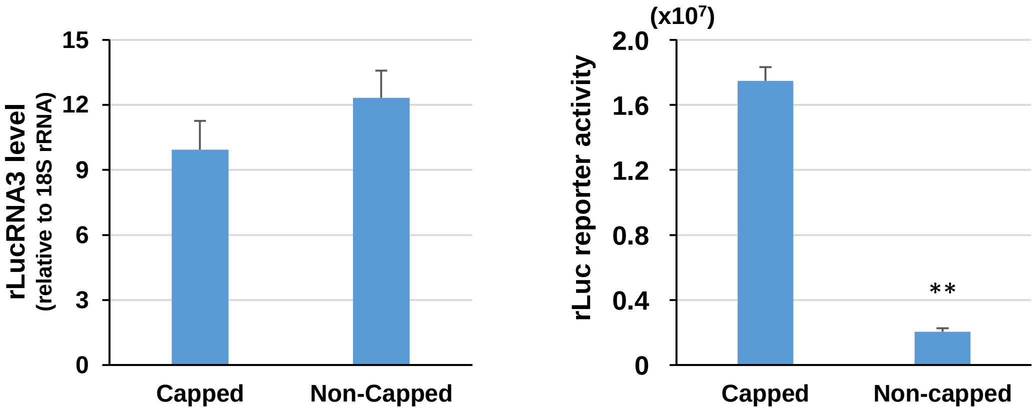

Fig. S4. Enhanced translation from expressed rLucRNA3 capped by vaccinia virus capping enzyme. 17Cl-1 cells were co-transfected with plasmids expressing T7 polymerase, rLucRNA3, vaccinia virus capping enzyme D1R and vaccinia virus capping enzyme D12L (capped). For the generation of uncapped rLucRNA3, the plasmids encoding vaccinia capping enzymes were omitted and pCAGGS-CAT was used to adjust the total amount of plasmids (uncapped). At 20 h post-transfection, the levels of rLucRNA3, relative to 18S rRNA (left panel), and rLuc reporter activities (right panel) were determined. Statistical analysis was done by non-paired *t*-test. ***p*<0.01.

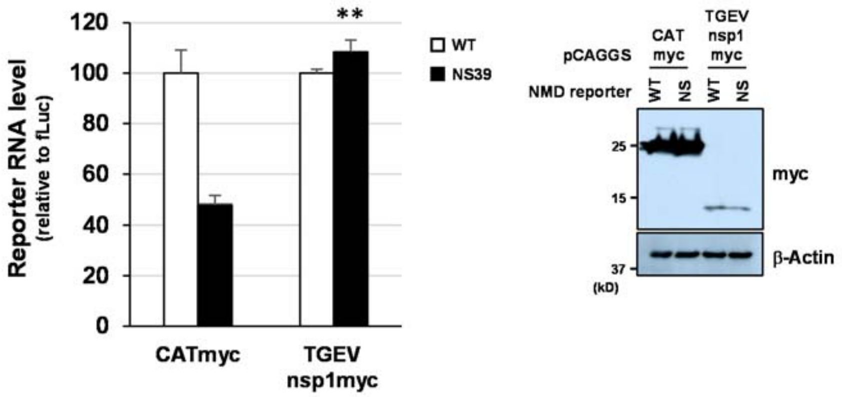

Fig. S5. An assay system to evaluate the inhibition of NMD pathway by expressed viral protein. 17Cl-1 cells were co-transfected with WT or NS39 reporter plasmid along with pCMV-fLuc plasmid, expressing fLuc transcripts, and a plasmid encoding myc-tagged CAT or myc-tagged TGEV nsp1. At 40 h post-transfection, the levels of WT reporter transcripts and NS39 reporter transcripts were determined by RT-qPCR and normalized to the levels of fLuc transcripts. NS39 transcript levels are shown relative to WT (set arbitrarily at 100). The data represent the mean with SEM of at least 3 independent experiments. Statistical analysis was done by ANOVA. **$p<0.01$. (left panel). Western blot analysis of CAT, TGEV nsp1 and β-actin at 40 h post-transfection (right panel).

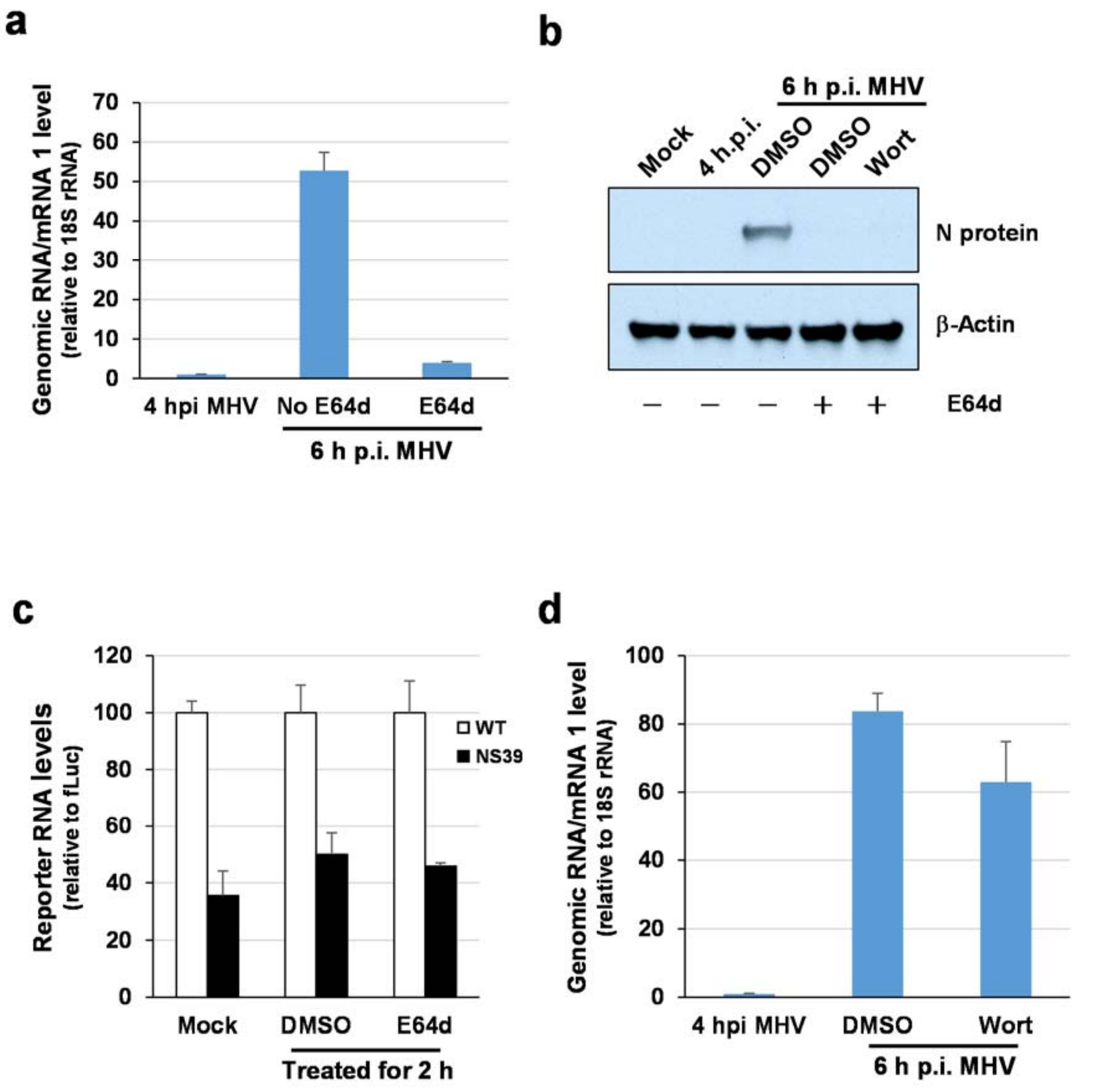

Fig. S6. Effect of wortmannin-mediated inhibition of NMD pathway on the accumulation of mRNA 1 in MHV-infected cells treated with E64d. (a) 17Cl-1 cells were infected with MHV at an m.o.i. of 0.1. Cells were incubated with 300 µg/mL of E64d or without E64d from 4 to 6 h p.i. The levels of mRNA 1, relative to 18S rRNA, at 4 and 6 h p.i. are shown. (b) 17Cl-1 cells were mock-infected (mock) or infected with MHV at an m.o.i. of 0.1. Cells were incubated with 300 µg/mL of E64d or left untreated from 4 h to 6 h p.i. The accumulation of N protein at 6 h p.i. was examined by Western blot analysis. (c) 17Cl-1 cells were co-transfected with NS39 reporter plasmid or WT reporter plasmid along with the pCMV-fLuc plasmid, expressing fLuc transcripts At 40 h post-transfection, total intracellular RNA was collected from one plate (Mock). Cells in another plate were incubated in the absence of E64d (DMSO) or treated with 300 µg/mL of E64d. After 2 h incubation with E64d, the levels of WT reporter transcripts and NS39 reporter transcripts were determined by RT-qPCR and normalized to the levels of fLuc transcripts. NS39 transcript levels are shown relative to WT (set arbitrarily at 100). (d)

17Cl-1 cells were infected with MHV at an m.o.i. of 0.1 and incubated in the presence of 20 μM of wortmannin or DMSO from 4 to 6 h p.i. The levels of mRNA 1, relative to 18S rRNA, at 4 and 6 h p.i. were determined by RT-qPCR. The data represent the mean with SEM of at least three independent experiments. Statistical analysis was done by ANOVA. $^*p<0.05$, $^{**}p<0.01$.

Table S1. List of primers for qPCR analysis

Primer name	Sequence	Expected size
rLuc-Fw	TGGGCCAGATGTAAACAAATGAATG	132 bp
rLuc-Rv	CACAACATGTCGCCATAAATAAGAAGAGG	
18S-Fw	CCGGTACAGTGAAACTGCGAATG	80 bp
18S-Rv	GTTATCCAAGTAGGAGAGGAGCGAG	
fLuc-Fw	CAGCTATTCTGATTACACCCGAG	97 bp
fLuc-Rv	CCGGTATCCAGATCCACAAC	
MHVleader-Fw	AAGAGTGATTGGCGTCCGTAC	
MHVmRNA 1-Rv	ATGGACACGTCACTGGCAGAG	174 bp
MHVmRNA 3-Rv	AAGAGTGATTGGCGTCCGTAC	142 bp
MHVmRNA 7-Rv	ATGGACACGTCACTGGCAGAG	173 bp
rpL3canonical-Fw	CTGAAGTTCATTGACACCACCTCCA	146 bp
rpL3canonical-Rv	ACCATCTGCACAAAGTGGTCCTG	
rpL3alternative-Fw	TCACCTGTTGCAAGGGTGATGAAG	155 bp
rpL3alternative-Rv	CTGTTGAAGTCCTTCTCCAGCTGC	

Fw: forward primer
Rv: reverse primer
MHVleader-Fw was used as the forward primer with each of the other reverse primers for MHV mRNAs.
